# The closedness of shift invariant subspaces in $L^{p,q} (\mathbb{R}^{d+1} )$

**DOI:** 10.1186/s13660-018-1755-2

**Published:** 2018-07-06

**Authors:** Qingyue Zhang

**Affiliations:** College of Science, Tianjin University of Technology, Tianjin, China

**Keywords:** 42C15, 42C40, 41A58, Mixed Lebesgue spaces $L^{p,q} (\mathbb{R}^{d+1} )$, Closedness of shift invariant subspaces, Shift invariant subspaces

## Abstract

In this paper, we consider the closedness of shift invariant subspaces in $L^{p,q} (\mathbb{R}^{d+1} )$. We first define the shift invariant subspaces generated by the shifts of finite functions in $L^{p,q} (\mathbb{R}^{d+1} )$. Then we give some necessary and sufficient conditions for the shift invariant subspaces in $L^{p,q} (\mathbb{R}^{d+1} )$ to be closed. Our results improve some known results in (Aldroubi et al. in J. Fourier Anal. Appl. **7**:1–21, 2001).

## Introduction and main result

$L^{p,q} (\mathbb{R}^{d+1} )$ ($1< p,q <+\infty$) are called mixed Lebesgue spaces which generalize Lebesgue spaces [2–6]. They are very important for the study of sampling and equation problems, since we can consider functions to be independent quantities with different properties [5–8]. Recently, Torres, Ward, Li, Liu and Zhang studied the sampling theorem on the shift invariant subspaces in $L^{p,q} (\mathbb{R}^{d+1} )$ [6–8]. In this environment, we study the closedness of shift invariant subspaces in $L^{p,q} (\mathbb{R}^{d+1} )$.

The closedness is an expected property for shift invariant subspaces, which is widely considered in the study of shift invariant subspaces. de Boor, DeVore, Ron, Bownik and Shen studied the closedness of shift invariant subspaces in $L^{2} (\mathbb{R}^{d} )$ [9–11]. And Jia, Micchelli, Aldroubi, Sun and Tang discussed the closedness of shift invariant subspaces in $L^{p} (\mathbb{R}^{d} )$ [1, 12, 13]. In this paper, we consider the closedness of shift invariant subspaces in $L^{p,q} (\mathbb{R}^{d+1} )$.

In order to provide our main result which extends the result in [1], we introduce some definitions and notations.

The definition of $L^{p,q} (\mathbb{R}^{d+1} )$ is as follows.

### Definition 1.1

For $1 < p,q <+\infty$. $L^{p,q}=L^{p,q} (\Bbb {R}^{d+1} )$ is made up of all functions *f* satisfying

$$\Vert f \Vert _{L^{p,q}}= \biggl[ \int_{\Bbb {R}} \biggl( \int _{\Bbb {R}^{d}} \bigl\vert f(x,y) \bigr\vert ^{q}\,dy \biggr)^{\frac {p}{q}}\,dx \biggr]^{\frac{1}{p}}< +\infty. $$

We define mixed sequence spaces $\ell^{p,q} (\mathbb {Z}^{d+1} )$ as follows:

$$\ell^{p,q}=\ell^{p,q} \bigl(\mathbb{Z}^{d+1} \bigr)= \biggl\{ c: \Vert c \Vert _{\ell^{p,q}}= \biggl[\sum _{n\in\Bbb {Z}} \biggl(\sum_{l \in\Bbb {Z}^{d}} \bigl\vert c(n,l) \bigr\vert ^{q} \biggr)^{\frac{p}{q}} \biggr]^{\frac{1}{p}} < +\infty \biggr\} . $$

Given a function *f*, define

$$\Vert f \Vert _{\mathcal{L}^{p,q}} := \biggl\Vert \sum _{k_{1}\in \mathbb{Z}} \biggl[ \int_{[0,1]^{d}}\biggl(\sum_{k_{2}\in \mathbb{Z}^{d}} \bigl\vert f(\cdot+k_{1},x_{2}+k_{2}) \bigr\vert \biggr)^{q}\,dx_{2} \biggr]^{1/q} \biggr\Vert _{L^{p}[0,1]}. $$

For $1\leq p,q\leq\infty$, let $\mathcal{L}^{p,q}=\mathcal{L}^{p,q} (\mathbb{R}^{d+1} )$ be the linear space of all functions *f* for which $\Vert f \Vert _{\mathcal{L}^{p,q}}<\infty$. The norms are defined above and with usual modification in the case of $p \mbox{ or } q=\infty$. $\mathcal{L}^{p,q}$ is a generalization of $\mathcal{L}^{p}$ (the definition of $\mathcal{L}^{p}$ see [14, Sect. 1]). Clearly, for $1\leq p,q \leq\infty$, one has $\mathcal{L}^{\infty ,\infty}\subset\mathcal{L}^{\infty}$ and $\mathcal{L}^{\infty,\infty}\subset\mathcal{L}^{p,q}\subset \mathcal{L}^{1,1}$.

Let $\hat{f}(\omega)$ denote the Fourier transform of $f\in L^{1} (\mathbb{R}^{d+1} )$:

$$\hat{f}(\omega)= \int_{\mathbb{R}^{d+1}}f(x)e^{-i\omega x}\,dx. $$

For a given sequence *c* and a function *ϕ*, $c\ast_{\mathrm {sd}}\phi=\sum_{k\in \mathbb{Z}^{d+1}}c(k)\phi(\cdot-k)$ is called semi-convolution of *c* and *ϕ*.

Assume that $\mathcal{B}$ is a Banach space. $(\mathcal{B})^{(r)}$ denotes *r* copies $\mathcal{B}\times\mathcal{B}\times\cdots\times \mathcal{B}$ of $\mathcal{B}$. If $C=(c_{1},c_{2},\ldots ,c_{r})^{T}\in(\mathcal{B})^{(r)}$, then one defines the norm of *C* by $\Vert C \Vert_{(\mathcal{B})^{(r)}}=\sum_{j=1}^{r} \Vert c_{j} \Vert_{\mathcal{B}}$.

$\mathcal{WC}^{p,q}$ ($1\leq p,q\leq\infty$) consists of all distributions whose Fourier coefficients belong to $\ell^{p,q}$. When $p=q=1$, $\mathcal{WC}^{1,1}$ becomes the Wiener class $\mathcal{WC}$.

Suppose that $\Theta=(\theta_{1},\theta_{2},\ldots,\theta _{r})^{T}$ and $\Psi=(\psi_{1},\psi_{2},\ldots,\psi_{s})^{T}$ are two vector functions which satisfy $\widehat{\theta}_{j}(\omega )\overline{\widehat{\psi}_{j'}(\omega)}$ ($1\leq j\leq r$, $1\leq j'\leq s$) are integrable. One defines

$$[\widehat{\Theta},\widehat{\Psi}](\omega)= \biggl(\sum _{k\in \mathbb{Z}^{d+1}}\widehat{\theta}_{j}(\omega+2k\pi)\overline{ \widehat{\psi }_{j'}(\omega+2k\pi)} \biggr)_{1\leq j\leq r,1\leq j'\leq s}. $$

### Remark 1.2

By [14, Theorem 3.1 and Theorem 3.2], $[\widehat{\Theta },\widehat{\Psi}](\omega)\in\mathcal{WC}$ for any $\Theta,\Psi \in\mathcal{L}^{\infty,\infty}\subset\mathcal{L}^{\infty}\subset \mathcal{L}^{2}$. Therefore, for any $\Theta\in\mathcal{L}^{\infty,\infty}$, using the continuity of $[\widehat{\Theta},\widehat{\Theta}](\omega)$ and $\operatorname {rank}[\widehat{\Theta},\widehat{\Theta}](\omega)= \operatorname{rank} (\widehat{\Theta}(\omega+2k\pi) )_{k\in \mathbb{Z}^{d+1}}$, one obtains, for any $n\geq0$, the set $\Omega _{n}= \{\omega:\operatorname{rank} (\widehat{\Theta }(\omega+2k\pi) ) _{k\in \mathbb{Z}^{d+1}}>n \}$ is open.

The following proposition shows that the shift invariant subspaces in $L^{p,q}$ ($1< p,q<\infty$) are well defined.

### Proposition 1.3

([8, Lemma 2.2])

*Let* $\theta\in\mathcal{L}^{p,q}$, *where* $1< p,q<\infty$. *Then*, *for any* $c\in\ell^{p,q}$,

$$\Vert c*_{\mathrm{sd}}\theta \Vert _{L^{p,q}}\leq \Vert c \Vert _{\ell^{p,q}} \Vert \theta \Vert _{\mathcal{L}^{p,q}}. $$

### Definition 1.4

For $\Theta=(\theta_{1},\theta_{2},\ldots,\theta_{r})^{T}\in (\mathcal{L}^{\infty,\infty})^{(r)}$, the multiply generated shift invariant subspace in the mixed Lebesgue spaces $L^{p,q}$ is defined by

$$\begin{aligned} V_{p,q}(\Theta)= \Biggl\{ \sum_{j=1}^{r} \sum_{k\in\Bbb {Z}^{d+1}}c_{j}(k)\theta_{j}( \cdot-k):c_{j}= \bigl\{ c_{j}(k):k\in\Bbb {Z}^{d+1} \bigr\} \in\ell^{p,q}, 1\leq j\leq r \Biggr\} . \end{aligned}$$

The following is our main result.

### Theorem 1.5

*Assume* $\Theta=(\theta_{1},\theta_{2},\ldots,\theta_{r})^{T}\in (\mathcal{L}^{\infty,\infty})^{(r)}$ *and* $1< p,q<\infty$. *Then the following four conditions are equivalent*.

(i) $V_{p,q}(\Theta)$ *is closed in* $L^{p,q}$.
(ii) *There exist some positive constants* $C_{1}$ *and* $C_{2}$ *satisfying*
  $$C_{1}[\widehat{\Theta},\widehat{\Theta}](\omega)\leq[\widehat { \Theta},\widehat{\Theta}](\omega)\overline{[\widehat{\Theta },\widehat{\Theta}]( \omega)^{T}} \leq C_{2}[\widehat{\Theta},\widehat{\Theta}]( \omega),\quad \forall \omega\in[-\pi,\pi]^{d+1}. $$
(iii) *There exist constants* $B_{1}, B_{2}>0$ *satisfying*
  $$B_{1} \Vert f \Vert _{L^{p,q}}\leq\inf_{f=\sum _{j=1}^{r}c_{j}*_{\mathrm{sd}}\phi_{j}} \sum_{j=1}^{r} \Vert c_{j} \Vert _{\ell^{p,q}} \leq B_{2} \Vert f \Vert _{L^{p,q}}, \quad\forall f\in V_{p,q}(\Theta). $$
(iv) *There is* $\Psi=(\psi_{1},\psi_{2},\ldots,\psi_{r})^{T}\in (\mathcal{L}^{\infty,\infty})^{(r)}$ *satisfying*
  $$\begin{aligned} f =&\sum_{j=1}^{r}\sum _{k\in \mathbb{Z}^{d+1}}\bigl\langle f,\psi_{j}(\cdot -k)\bigr\rangle \theta_{j}(\cdot-k) \\ =&\sum_{j=1}^{r}\sum _{k\in \mathbb{Z}^{d+1}}\bigl\langle f,\theta_{j}(\cdot -k)\bigr\rangle \psi_{j}(\cdot-k),\quad\forall f\in V_{p,q}(\Theta). \end{aligned}$$

The paper is organized as follows. In the next section, we give some three useful lemmas and two propositions. In Sect. 3, we give the proof of Theorem 1.5. Finally, concluding remarks are presented in Sect. 4.

## Some useful lemmas and propositions

In this section, we give three useful lemmas and two propositions which are needed in the proof of Theorem 1.5.

### Proposition 2.1

([1, Lemma 1])

*Let* $\Theta\in(\mathcal{L}^{2})^{(r)}$. *Then the following are equivalent*:

(i) $\operatorname{rank} (\widehat{\Theta}(\omega+2k\pi ) )_{k\in \mathbb{Z}^{d+1}}$ *is a constant for any* $\omega\in \mathbb{R}^{d+1}$.
(ii) *There exist some positive constants* $C_{1}$ *and* $C_{2}$ *such that*
  $$C_{1}[\widehat{\Theta},\widehat{\Theta}](\omega)\leq[\widehat { \Theta},\widehat{\Theta}](\omega)\overline{[\widehat{\Theta },\widehat{\Theta}]( \omega)^{T}} \leq C_{2}[\widehat{\Theta},\widehat{\Theta}]( \omega),\quad \forall \omega\in[-\pi,\pi]^{d+1}. $$

### Proposition 2.2

([1, Lemma 2])

*Let* $\Phi\in(\mathcal{L}^{2})^{(r)}$ *satisfy* $\operatorname {rank} (\widehat{\Phi}(\xi+2k\pi) ) _{k\in \mathbb{Z}^{d+1}}=k_{0} \geq1$ *for all* $\xi\in \mathbb{R}^{d+1}$. *Then there exists a finite index set* Λ, $\eta_{\lambda}\in[-\pi,\pi]^{d+1}$, $0<\delta_{\lambda}<1/4$, *nonsingular* 2*π*-*periodic* $r\times r$ *matrix* $P_{\lambda}(\xi)$ *with all entries in the Wiener class and* $K_{\lambda} \subset \mathbb{Z}^{d+1}$ *with* $\operatorname{cardinality}(K_{\lambda})= k_{0}$ *for all* $\lambda\in\Lambda$, *having the following properties*:

(i) $$[-\pi,\pi]^{d+1}\subset\bigcup_{\lambda\in\Lambda}B(\delta _{\lambda}, \delta_{\lambda}/2), $$
  *where* $B(x_{0}, \delta)$ *denotes the open ball in* $\mathbb{R}^{d+1}$ *with center* $x_{0}$ *and radius* *δ*;
(ii) $$P_{\lambda }(\xi )\hat{\Phi }(\xi )=\left(\begin{array}{c} {\hat{\Psi }}_{1,\lambda }(\xi )\\ {\hat{\Psi }}_{2,\lambda }(\xi ) \end{array}\right),\;\xi \in R^{d+1}\text{ and }\lambda \in \Lambda ,$$
  *where* $\Psi_{1,\lambda}$ *and* $\Psi_{2,\lambda}$ *are functions from* $\mathbb{R}^{d+1}$ *to* $\mathbb{C}^{k_{0}}$ *and* $\mathbb{C}^{r-k_{0}}$, *respectively*, *satisfying*
  $$\operatorname{rank} \bigl(\widehat{\Psi}_{1,\lambda}(\xi+2\pi k) \bigr)_{k\in K_{\lambda} } =k_{0},\quad\forall \xi\in B(\delta _{\lambda},\delta_{\lambda}/2) $$
  *and*
  $$\widehat{\Psi}_{2,\lambda}(\xi)=0,\quad\forall \xi\in B(\delta _{\lambda},8\delta_{\lambda}/5)+2\pi \mathbb{Z}^{d+1}. $$

*Furthermore*, *there exist* 2*π*-*periodic* $\mathbb{C}^{\infty}$ *functions* $h_{\lambda}(\xi)$, $\lambda\in\Lambda$, *on* $\mathbb{R}^{d+1}$ *such that*

$$\begin{aligned} \sum_{\lambda\in\Lambda}h_{\lambda}(\xi)=1,\quad\forall \xi \in \mathbb{R}^{d+1} \end{aligned}$$

*and*

$$\begin{aligned} \operatorname{supp}h_{\lambda}(\xi)\subset B(\delta_{\lambda }, \delta_{\lambda}/2)+2\pi \mathbb{Z}^{d+1}. \end{aligned}$$

The following lemma can be proved similarly to [7, Theorem 3.4]. And we leave the details to the interested reader.

### Lemma 2.3

*Assume that* $f\in L^{p,q}$ ($1< p,q<\infty$) *and* $g\in\mathcal {L}^{\infty,\infty}$. *Then*

$$\biggl\Vert \biggl\{ \int_{\mathbb{R}} \int_{\mathbb{R}^{d}}f(x_{1},x_{2})\overline {g(x_{1}-k_{1},x_{2}-k_{2})}\,dx_{1}\,dx_{2}:k_{1} \in \mathbb{Z},k_{2}\in \mathbb{Z}^{d} \biggr\} \biggr\Vert _{\ell^{p,q}}\leq \Vert f \Vert _{L^{p,q}} \Vert g \Vert _{\mathcal{L}^{\infty,\infty}}. $$

### Lemma 2.4

*Let* $c\in\ell^{1}$. *Then one has*:

(i) *If* $\theta\in\mathcal{L}^{p,q}$ ($1< p,q< \infty$), *then*
  $$\Vert c*_{\mathrm{sd}}\theta \Vert _{\mathcal {L}^{p,q}}\leq \Vert c \Vert _{\ell^{1}} \Vert \theta \Vert _{\mathcal{L}^{p,q}}. $$
(ii) *If* $\theta\in\mathcal{L}^{\infty,\infty}$, *then*
  $$\Vert c*_{\mathrm{sd}}\theta \Vert _{\mathcal{L}^{\infty ,\infty}}\leq \Vert c \Vert _{\ell^{1}} \Vert \theta \Vert _{\mathcal{L}^{\infty,\infty}}. $$

### Proof

(i) By Young’s inequality and the triangle inequality, one has

$$\begin{aligned} \Vert c*_{\mathrm{sd}}\theta \Vert _{\mathcal {L}^{p,q}} =& \biggl\Vert \sum _{n\in \mathbb{Z}} \biggl[ \int _{[0,1]^{d}} \biggl(\sum_{l\in \mathbb{Z}^{d}} \bigl\vert c*_{\mathrm {sd}}\theta(\cdot+n,y+l) \bigr\vert \biggr)^{q}\,dy \biggr]^{1/q} \biggr\Vert _{L^{p}[0,1]} \\ =& \biggl\Vert \sum_{n\in \mathbb{Z}} \biggl[ \int_{[0,1]^{d}} \biggl(\sum_{l\in \mathbb{Z}^{d}} \biggl\vert \sum_{n'\in \mathbb{Z}}\sum_{l'\in \mathbb{Z}^{d}} c_{n',l'}\theta\bigl(\cdot+n-n',y+l-l'\bigr) \biggr\vert \biggr)^{q}\,dy \biggr]^{1/q} \biggr\Vert _{L^{p}[0,1]} \\ \leq& \biggl\Vert \sum_{n\in \mathbb{Z}} \biggl[ \int_{[0,1]^{d}} \biggl(\sum_{n'\in \mathbb{Z}}\sum _{l\in \mathbb{Z}^{d}} \biggl\vert \sum _{l'\in \mathbb{Z}^{d}} c_{n',l'}\theta\bigl(\cdot+n-n',y+l-l' \bigr) \biggr\vert \biggr)^{q}\,dy \biggr]^{1/q} \biggr\Vert _{L^{p}[0,1]} \\ \leq& \biggl\Vert \sum_{n\in \mathbb{Z}} \biggl[ \int_{[0,1]^{d}} \biggl(\sum_{n'\in \mathbb{Z}}\sum _{l\in \mathbb{Z}^{d}} \vert c_{n',l} \vert \biggl(\sum _{l'\in \mathbb{Z}^{d}} \bigl\vert \theta\bigl(\cdot+n-n',y+l' \bigr) \bigr\vert \biggr) \biggr)^{q}\,dy \biggr]^{1/q} \biggr\Vert _{L^{p}[0,1]} \\ \leq& \biggl\Vert \sum_{n\in \mathbb{Z}}\sum _{n'\in \mathbb{Z}}\sum_{l\in \mathbb{Z}^{d}} \vert c_{n',l} \vert \biggl[ \int _{[0,1]^{d}} \biggl(\sum_{l'\in \mathbb{Z}^{d}} \bigl\vert \theta\bigl(\cdot+n-n',y+l'\bigr) \bigr\vert \biggr)^{q}\,dy \biggr]^{1/q} \biggr\Vert _{L^{p}[0,1]} \\ \leq& \biggl\Vert \sum_{n'\in \mathbb{Z}}\sum _{l\in \mathbb{Z}^{d}} \vert c_{n',l} \vert \sum _{n\in \mathbb{Z}} \biggl[ \int _{[0,1]^{d}} \biggl(\sum_{l'\in \mathbb{Z}^{d}} \bigl\vert \theta\bigl(\cdot+n-n',y+l'\bigr) \bigr\vert \biggr)^{q}\,dy \biggr]^{1/q} \biggr\Vert _{L^{p}[0,1]} \\ \leq& \sum_{n'\in \mathbb{Z}}\sum_{l\in \mathbb{Z}^{d}} \vert c_{n',l} \vert \biggl\Vert \sum_{n\in \mathbb{Z}} \biggl[ \int _{[0,1]^{d}} \biggl(\sum_{l'\in \mathbb{Z}^{d}} \bigl\vert \theta\bigl(\cdot+n-n',y+l'\bigr) \bigr\vert \biggr)^{q}\,dy \biggr]^{1/q} \biggr\Vert _{L^{p}[0,1]} \\ \leq&\sum_{n'\in \mathbb{Z}}\sum_{l\in \mathbb{Z}^{d}} \vert c_{n',l} \vert \biggl\Vert \sum_{n\in \mathbb{Z}} \biggl[ \int _{[0,1]^{d}} \biggl(\sum_{l'\in \mathbb{Z}^{d}} \bigl\vert \theta\bigl(\cdot+n,y+l'\bigr) \bigr\vert \biggr)^{q}\,dy \biggr]^{1/q} \biggr\Vert _{L^{p}[0,1]} \\ =& \Vert c \Vert _{\ell^{1}} \Vert \theta \Vert _{\mathcal{L}^{p,q}}. \end{aligned}$$

The desired result (i) in Lemma 2.4 is obtained.

(ii) The desired result (ii) in Lemma 2.4 can be found in [8, Lemma 2.4]. □

### Lemma 2.5

*Assume that* $\theta\in\mathcal{L}^{p,q}$ ($1< p,q<\infty$) *and* $\sum_{k\in \mathbb{Z}^{d+1}}\theta(\cdot-k)=0$. *Then for any function* *h* *on* $\mathbb{R}^{d+1}$ *satisfying*

2.1 $$ \bigl\vert h(x) \bigr\vert \leq D\bigl(1+ \vert x \vert \bigr)^{-d-2}\quad\textit{and} \quad \bigl\vert h(x)-h(y) \bigr\vert \leq D \vert x-y \vert \bigl(1+\min\bigl( \vert x \vert , \vert y \vert \bigr)\bigr)^{-d-2}, $$

*one has*

$$\lim_{n\rightarrow\infty}2^{-n(d+1)} \biggl\Vert \sum _{k\in \mathbb{Z}^{d+1}}h\bigl(2^{-n}k\bigr)\theta(\cdot-k) \biggr\Vert _{\mathcal{L}^{p,q}}=0. $$

*Here* *D* *in* (2.1) *is a positive constant*.

### Proof

Since $\theta\in\mathcal{L}^{p,q}$, for any $\varepsilon> 0$, there is $N_{0}\geq2$ satisfying

2.2 $$\begin{aligned} \biggl\Vert \sum_{ \vert l \vert \geq N_{0}} \biggl( \int _{[0,1]^{d}} \biggl(\sum_{k\in \mathbb{Z}^{d}} \bigl\vert \theta(\cdot +l,x+k) \bigr\vert \biggr)^{q}\,dx \biggr)^{1/q} \biggr\Vert _{L^{p}[0,1]}< \epsilon \end{aligned}$$

and

2.3 $$\begin{aligned} \biggl\Vert \sum_{l\in \mathbb{Z}} \biggl( \int_{[0,1]^{d}} \biggl(\sum_{k\in E^{d}_{N_{0}}} \bigl\vert \theta(\cdot+l,x+k) \bigr\vert \biggr)^{q}\,dx \biggr)^{1/q} \biggr\Vert _{L^{p}[0,1]}< \epsilon, \end{aligned}$$

where $E^{d}_{N_{0}}=\{(k_{1},\ldots,k_{d}): \mbox{ there exists some } 1\leq i_{0}\leq \,d \mbox{ such that } |k_{i_{0}}|>N_{0}\}$.

Set

$$\begin{aligned} \theta_{1}(x_{1},\ldots,x_{d+1}) =& \theta(x_{1},\ldots,x_{d+1})\chi _{O_{N_{0}}}(x_{1}, \ldots,x_{d+1}) \\ &{}+\sum_{(k_{1},\ldots,k_{d+1})\in E^{d+1}_{N_{0}}}\theta (x_{1}+k_{1}, \ldots,x_{d+1}+k_{d+1})\chi_{[0,1]^{d+1}}(x_{1}, \ldots,x_{d+1}), \end{aligned}$$

where $O_{N_{0}}=\bigcup_{|k_{i}|\leq N_{0}, 1\leq i\leq d+1} [(k_{1},\ldots,k_{d+1})+[0,1]^{d+1} ]$ and $\chi_{S}$ is the characteristic function of *S*.

Thus $\sum_{k\in \mathbb{Z}^{d+1}}\theta_{1}(\cdot-k)=\sum_{k\in \mathbb{Z}^{d+1}}\theta(\cdot-k)=0$ and $\Vert\theta_{1}-\theta\Vert _{\mathcal{L}^{p,q}}<5\epsilon$. In fact

$$\begin{aligned} & \Vert \theta_{1}-\theta \Vert _{\mathcal{L}^{p,q}} \\ &\quad= \biggl\Vert \sum_{l\in \mathbb{Z}} \biggl( \int_{[0,1]^{d}} \biggl(\sum_{k\in \mathbb{Z}^{d}} \bigl\vert (\theta_{1}-\theta) (\cdot +l,x+k) \bigr\vert \biggr)^{q}\,dx \biggr)^{1/q} \biggr\Vert _{L^{p}[0,1]} \\ &\quad\leq \biggl\Vert \biggl( \int_{[0,1]^{d}} \biggl(\sum_{k\in \mathbb{Z}^{d}} \bigl\vert (\theta_{1}-\theta) (\cdot,x+k) \bigr\vert \biggr)^{q}\,dx \biggr)^{1/q} \biggr\Vert _{L^{p}[0,1]} \\ &\qquad{}+ \biggl\Vert \sum_{l\neq0} \biggl( \int_{[0,1]^{d}} \biggl(\sum_{k\in \mathbb{Z}^{d}} \bigl\vert (\theta_{1}-\theta) (\cdot +l,x+k) \bigr\vert \biggr)^{q}\,dx \biggr)^{1/q} \biggr\Vert _{L^{p}[0,1]} \\ &\quad=I_{1}+I_{2}. \end{aligned}$$

First of all, one treats $I_{1}$: by (2.2) and (2.3), one has

$$\begin{aligned} I_{1} \leq& \biggl\Vert \biggl( \int_{[0,1]^{d}} \bigl( \bigl\vert (\theta_{1}-\theta) ( \cdot,x) \bigr\vert \bigr)^{q}\,dx \biggr)^{1/q} \\ &{}+ \biggl( \int_{[0,1]^{d}} \biggl(\sum_{k\neq0} \bigl\vert (\theta_{1}-\theta) (\cdot,x+k) \bigr\vert \biggr)^{q}\,dx \biggr)^{1/q} \biggr\Vert _{L^{p}[0,1]} \\ \leq& \biggl\Vert \biggl( \int_{[0,1]^{d}} \bigl( \bigl\vert (\theta _{1}-\theta) ( \cdot,x) \bigr\vert \bigr)^{q}\,dx \biggr)^{1/q} \biggr\Vert _{L^{p}[0,1]} \\ &{}+ \biggl\Vert \biggl( \int_{[0,1]^{d}} \biggl(\sum_{k\neq0} \bigl\vert (\theta_{1}-\theta) (\cdot,x+k) \bigr\vert \biggr)^{q}\,dx \biggr)^{1/q} \biggr\Vert _{L^{p}[0,1]} \\ \leq& \biggl\Vert \biggl( \int_{[0,1]^{d}} \biggl(\sum_{(k_{1},\ldots ,k_{d+1})\in E^{d+1}_{N_{0}}} \bigl\vert \theta(\cdot+k_{1},\ldots ,x_{d+1}+k_{d+1}) \bigr\vert \biggr)^{q}\,dx \biggr)^{1/q} \biggr\Vert _{L^{p}[0,1]} \\ &{}+ \biggl\Vert \biggl( \int_{[0,1]^{d}} \biggl(\sum_{k\in E^{d}_{N_{0}}} \bigl\vert \theta(\cdot,x+k) \bigr\vert \biggr)^{q}\,dx \biggr)^{1/q} \biggr\Vert _{L^{p}[0,1]} \\ \leq& \biggl\Vert \biggl( \int_{[0,1]^{d}} \biggl( \biggl(\sum_{ \vert l \vert >N_{0}, k\in \mathbb{Z}^{d}}+ \sum_{l\in \mathbb{Z}, k\in E^{d}_{N_{0}}} \biggr) \bigl\vert \theta(\cdot+l,x+k) \bigr\vert \biggr)^{q}\,dx \biggr)^{1/q} \biggr\Vert _{L^{p}[0,1]} \\ &{}+ \biggl\Vert \sum_{l\in \mathbb{Z}} \biggl( \int_{[0,1]^{d}} \biggl(\sum_{k\in E^{d}_{N_{0}}} \bigl\vert \theta(\cdot+l,x+k) \bigr\vert \biggr)^{q}\,dx \biggr)^{1/q} \biggr\Vert _{L^{p}[0,1]} \\ \leq& \biggl\Vert \biggl( \int_{[0,1]^{d}} \biggl(\sum_{ \vert l \vert >N_{0}, k\in \mathbb{Z}^{d}} \bigl\vert \theta(\cdot+l,x+k) \bigr\vert \biggr)^{q}\,dx \biggr)^{1/q} \biggr\Vert _{L^{p}[0,1]} \\ &{}+ \biggl\Vert \biggl( \int_{[0,1]^{d}} \biggl(\sum_{l\in \mathbb{Z}, k\in E^{d}_{N_{0}}} \bigl\vert \theta(\cdot+l,x+k) \bigr\vert \biggr)^{q}\,dx \biggr)^{1/q} \biggr\Vert _{L^{p}[0,1]}+\epsilon \\ \leq& \biggl\Vert \sum_{ \vert l \vert >N_{0}} \biggl( \int_{[0,1]^{d}} \biggl(\sum_{ k\in \mathbb{Z}^{d}} \bigl\vert \theta (\cdot+l,x+k) \bigr\vert \biggr)^{q}\,dx \biggr)^{1/q} \biggr\Vert _{L^{p}[0,1]} \\ &{}+ \biggl\Vert \sum_{l\in \mathbb{Z}} \biggl( \int_{[0,1]^{d}} \biggl(\sum_{ k\in E^{d}_{N_{0}}} \bigl\vert \theta(\cdot+l,x+k) \bigr\vert \biggr)^{q}\,dx \biggr)^{1/q} \biggr\Vert _{L^{p}[0,1]}+\epsilon \\ < &\epsilon+\epsilon+\epsilon=3\epsilon. \end{aligned}$$

Next, one treats $I_{2}$:

$$\begin{aligned} I_{2} \leq& \biggl\Vert \sum_{ \vert l \vert >N_{0}} \biggl( \int_{[0,1]^{d}} \biggl(\sum_{k\in \mathbb{Z}^{d}} \bigl\vert (\theta _{1}-\theta) (\cdot+l,x+k) \bigr\vert \biggr)^{q}\,dx \biggr)^{1/q} \biggr\Vert _{L^{p}[0,1]} \\ &{}+ \biggl\Vert \sum_{ \vert l \vert \leq N_{0}, l\neq 0} \biggl( \int_{[0,1]^{d}} \biggl(\sum_{k\in \mathbb{Z}^{d}} \bigl\vert (\theta_{1}-\theta) (\cdot+l,x+k) \bigr\vert \biggr)^{q}\,dx \biggr)^{1/q} \biggr\Vert _{L^{p}[0,1]} \\ =& \biggl\Vert \sum_{ \vert l \vert >N_{0}} \biggl( \int _{[0,1]^{d}} \biggl(\sum_{k\in \mathbb{Z}^{d}} \bigl\vert \theta (\cdot+l,x+k) \bigr\vert \biggr)^{q}\,dx \biggr)^{1/q} \biggr\Vert _{L^{p}[0,1]} \\ &{}+ \biggl\Vert \sum_{ \vert l \vert \leq N_{0}, l\neq 0} \biggl( \int_{[0,1]^{d}} \biggl(\sum_{k\in E^{d}_{N_{0}}} \bigl\vert \theta(\cdot+l,x+k) \bigr\vert \biggr)^{q}\,dx \biggr)^{1/q} \biggr\Vert _{L^{p}[0,1]} \\ \leq& \biggl\Vert \sum_{ \vert l \vert >N_{0}} \biggl( \int_{[0,1]^{d}} \biggl(\sum_{k\in \mathbb{Z}^{d}} \bigl\vert \theta (\cdot+l,x+k) \bigr\vert \biggr)^{q}\,dx \biggr)^{1/q} \biggr\Vert _{L^{p}[0,1]} \\ &{}+ \biggl\Vert \sum_{l\in \mathbb{Z}} \biggl( \int_{[0,1]^{d}} \biggl(\sum_{k\in E^{d}_{N_{0}}} \bigl\vert \theta(\cdot+l,x+k) \bigr\vert \biggr)^{q}\,dx \biggr)^{1/q} \biggr\Vert _{L^{p}[0,1]} \\ < &\epsilon+\epsilon=2\epsilon. \end{aligned}$$

Therefore, one has $\Vert\theta_{1}-\theta\Vert_{\mathcal {L}^{p,q}}<5\epsilon$.

Using Lemma 2.4 and (2.1), there exists some positive constant *C* such that

$$\begin{aligned} & \biggl\Vert 2^{-n(d+1)}\sum_{k\in \mathbb{Z}^{d+1}}h \bigl(2^{-n}k\bigr) \bigl(\phi(\cdot -k)-\phi_{1}(\cdot-k) \bigr) \biggr\Vert _{\mathcal{L}^{p,q}} \\ &\quad\leq2^{-n(d+1)}\sum_{k\in \mathbb{Z}^{d+1}} \bigl\vert h \bigl(2^{-n}k\bigr) \bigr\vert \Vert \phi_{1}-\phi \Vert _{\mathcal {L}^{p,q}}\leq C\epsilon. \end{aligned}$$

Thus

$$\begin{aligned} & \biggl\Vert 2^{-n(d+1)}\sum_{k\in \mathbb{Z}^{d+1}}h \bigl(2^{-n}k\bigr)\theta _{1}(\cdot-k) \biggr\Vert _{\mathcal{L}^{p,q}} \\ &\quad=2^{-n(d+1)} \biggl\Vert \sum_{j_{1}\in \mathbb{Z}} \biggl( \int _{[0,1]^{d}} \biggl(\sum_{j_{2}\in \mathbb{Z}^{d}} \biggl\vert \sum_{k_{1}\in \mathbb{Z},k_{2}\in \mathbb{Z}^{d}}h \bigl(2^{-n}k_{1},2^{-n}k_{2}\bigr)\\ &\qquad \times{} \theta_{1}(\cdot +j_{1}-k_{1},x_{2}+j_{2}-k_{2}) \biggr\vert \biggr)^{q}\,dx_{2} \biggr)^{1/q} \biggr\Vert _{L^{p}[0,1]} \\ &\quad=2^{-n(d+1)} \biggl\Vert \sum_{j_{1}\in \mathbb{Z}} \biggl( \int _{[0,1]^{d}} \biggl(\sum_{j_{2}\in \mathbb{Z}^{d}} \biggl\vert \sum_{k_{1}\in \mathbb{Z},k_{2}\in \mathbb{Z}^{d}}\bigl(h\bigl(2^{-n}k_{1},2^{-n}k_{2} \bigr)-h\bigl(2^{-n}j_{1},2^{-n}j_{2} \bigr)\bigr) \\ &\qquad{}\times\theta_{1}(\cdot+j_{1}-k_{1},x_{2}+j_{2}-k_{2}) \biggr\vert \biggr)^{q}\,dx_{2}\biggr)^{1/q} \biggr\Vert _{L^{p}[0,1]} \\ &\quad\leq2^{-n(d+2)}C_{1}(N_{0}) \biggl\Vert \sum _{j_{1}\in \mathbb{Z}} \biggl( \int_{[0,1]^{d}} \biggl(\sum_{j_{2}\in \mathbb{Z}^{d}}\sum _{k_{1}\in \mathbb{Z},k_{2}\in \mathbb{Z}^{d}} \bigl(1+2^{-n} \bigl\vert (k_{1},k_{2}) \bigr\vert \bigr)^{-(d+2)}\\ &\qquad {}\times \bigl\vert \theta_{1}(\cdot +j_{1}-k_{1},x_{2}+j_{2}-k_{2}) \bigr\vert \biggr)^{q}\,dx_{2}\biggr)^{1/q} \biggr\Vert _{L^{p}[0,1]} \\ &\quad=2^{-n(d+2)}C_{1}(N_{0}) \biggl\Vert \sum _{j_{1}\in \mathbb{Z}} \biggl( \int_{[0,1]^{d}} \biggl(\sum_{k_{1}\in \mathbb{Z}} \sum_{k_{2}\in \mathbb{Z}^{d}} \bigl(1+2^{-n} \bigl\vert (k_{1},k_{2}) \bigr\vert \bigr)^{-(d+2)}\\ &\qquad {}\times\sum_{j_{2}\in \mathbb{Z}^{d}} \bigl\vert \theta_{1}(\cdot+j_{1}-k_{1},x_{2}+j_{2}) \bigr\vert \biggr)^{q}\,dx_{2} \biggr)^{1/q} \biggr\Vert _{L^{p}[0,1]} \\ &\quad\leq2^{-n(d+2)}C_{1}(N_{0})\sum _{k_{1}\in \mathbb{Z},k_{2}\in \mathbb{Z}^{d}} \bigl(1+2^{-n} \bigl\vert (k_{1},k_{2}) \bigr\vert \bigr)^{-(d+2)} \\ &\qquad{}\times\biggl\Vert \sum_{j_{1}\in \mathbb{Z}} \biggl( \int _{[0,1]^{d}} \biggl(\sum_{j_{2}\in \mathbb{Z}^{d}} \bigl\vert \theta_{1}(\cdot+j_{1}-k_{1},x_{2}+j_{2}) \bigr\vert \biggr)^{q}\,dx_{2} \biggr)^{1/q} \biggr\Vert _{L^{p}[0,1]} \\ &\quad\leq2^{-n}C_{2}(N_{0}) \biggl\Vert \sum _{j_{1}\in \mathbb{Z}} \biggl( \int_{[0,1]^{d}} \biggl(\sum_{j_{2}\in \mathbb{Z}^{d}} \bigl\vert \theta _{1}(\cdot+j_{1},x_{2}+j_{2}) \bigr\vert \biggr)^{q}\,dx_{2} \biggr)^{1/q} \biggr\Vert _{L^{p}[0,1]} \\ &\quad=2^{-n}C_{2}(N_{0}) \Vert \theta_{1} \Vert _{\mathcal{L}^{p,q}}\leq2^{-n}C_{2}(N_{0}) \bigl( \Vert \theta \Vert _{\mathcal{L}^{p,q}}+5\epsilon\bigr). \end{aligned}$$

Here $C_{i}(N_{0})$ ($i = 1,2$) are positive constants depending only on $N_{0}$ and *d*. This completes the proof. □

## Proof of Theorem 1.5

In this section, we give the proof of Theorem 1.5. The main steps of the proof are as follows: $\mbox{(iv)}\Rightarrow \mbox{(iii)}\Rightarrow\mbox{(i)}\Rightarrow\mbox{(ii)}\Rightarrow\mbox{(iv)}$.

$\mbox{(iv)}\Rightarrow\mbox{(iii)}$:

Let $f=\sum_{j=1}^{r}\sum_{k\in \mathbb{Z}^{d+1}}\langle f,\psi_{j}(\cdot -k)\rangle\theta_{j}(\cdot-k)$. Then, by Lemma 2.3, one has

$$\begin{aligned} \inf_{f=\sum_{j=1}^{r}c_{j}*_{\mathrm{sd}}\phi_{j}}\sum_{j=1}^{r} \Vert c_{j} \Vert _{\ell^{p,q}} \leq&\sum _{j=1}^{r} \bigl\Vert \bigl\{ \bigl\langle f, \psi_{j}(\cdot-k_{1},\cdot -k_{2})\bigr\rangle : k_{1}\in \mathbb{Z},k_{2}\in \mathbb{Z}^{d} \bigr\} \bigr\Vert _{\ell^{p,q}} \\ \leq&\sum_{j=1}^{r} \Vert f \Vert _{L^{p,q}} \Vert \psi_{j} \Vert _{\mathcal{L}^{\infty,\infty}}= \Vert f \Vert _{L^{p,q}}\sum_{j=1}^{r} \Vert \psi_{j} \Vert _{\mathcal{L}^{\infty,\infty}}. \end{aligned}$$

Conversely, if $f=\sum_{j=1}^{r}c_{j}*_{\mathrm{sd}}\theta_{j}$, then, by Proposition 1.3 and the triangle inequality

3.1 $$\begin{aligned} \Vert f \Vert _{L^{p,q}} =& \Biggl\Vert \sum _{j=1}^{r}c_{j}*_{\mathrm{sd}} \theta_{j} \Biggr\Vert _{L^{p,q}}\leq \sum _{j=1}^{r} \Vert c_{j}*_{\mathrm{sd}} \theta_{j} \Vert _{L^{p,q}} \\ \leq& \sum_{j=1}^{r} \Vert c_{j} \Vert _{\ell^{p,q}} \Vert \theta_{j} \Vert _{\mathcal{L}^{p,q}}\leq\max_{1\leq j\leq r} \Vert \theta_{j} \Vert _{\mathcal {L}^{p,q}}\sum_{j=1}^{r} \Vert c_{j} \Vert _{\ell^{p,q}}. \end{aligned}$$

Taking the infimum for (3.1), one gets

$$\Vert f \Vert _{L^{p,q}}\leq\max_{1\leq j\leq r} \Vert \theta_{j} \Vert _{\mathcal{L}^{p,q}}\inf_{f=\sum _{j=1}^{r}c_{j}*_{\mathrm{sd}}\theta_{j}}\sum _{j=1}^{r} \Vert c_{j} \Vert _{\ell^{p,q}}. $$

Let $B_{1}=1/\max_{1\leq j\leq r} \Vert\theta_{j} \Vert_{\mathcal {L}^{p,q}}$ and $B_{2}=\sum_{j=1}^{r} \Vert\psi_{j} \Vert_{\mathcal {L}^{\infty,\infty}}$. Then one has

$$B_{1} \Vert f \Vert _{L^{p,q}}\leq\inf_{f=\sum _{j=1}^{r}c_{j}*_{\mathrm{sd}}\phi_{j}} \sum_{j=1}^{r} \Vert c_{j} \Vert _{\ell^{p,q}} \leq B_{2} \Vert f \Vert _{L^{p,q}}, \quad\forall f\in V_{p,q}(\Theta). $$

$\mbox{(iii)}\Rightarrow\mbox{(i)}$:

For convenience, let $T:(\ell^{p,q})^{(r)}\rightarrow V_{p,q}(\Theta )$ be a mapping which is defined by

$$TC=\sum_{j=1}^{r}c_{j}*_{\mathrm{sd}} \theta_{j},\quad C=(c_{1},c_{2}, \ldots,c_{r})^{T}\in\bigl(\ell^{p,q} \bigr)^{(r)}, $$

and let $\Vert f \Vert_{\mathrm{inf}}=\inf_{f=\sum _{j=1}^{r}c_{j}*_{\mathrm{sd}}\theta_{j}}\sum_{j=1}^{r} \Vert c_{j} \Vert_{\ell^{p,q}}$. Then, obviously, $\Vert \cdot\Vert_{\mathrm {inf}}$ is a norm. Assume $f_{n}\subset\operatorname{Ran}(T)$ ($n\geq1$) is a Cauchy sequence. Here $\operatorname{Ran}(T)$ denotes the range of *T*. Without loss of generality, let $\Vert f_{n}-f_{n-1} \Vert_{\mathrm {inf}}<2^{-n}$. Using the definition of $\Vert\cdot\Vert_{\mathrm{inf}}$, there is $C_{n}\in(\ell ^{p,q})^{(r)}$ ($n\geq2$) such that $TC_{n}=f_{n}-f_{n-1}$ and $\Vert C_{n} \Vert_{(\ell^{p,q})^{(r)}}<2^{-n}$ for any $n\geq2$. By the completeness of $(\ell^{p,q})^{(r)}$ and $\sum_{n=2}^{\infty} \Vert C_{n} \Vert_{(\ell^{p,q})^{(r)}}<\infty$, one has $Z=\sum_{n=2}^{\infty}C_{n}\in(\ell^{p,q})^{(r)}$ and $f_{1}+TZ\in \operatorname{Ran}(T)$. Note that $\Vert TC \Vert_{\mathrm{inf}}\leq \Vert C \Vert_{(\ell ^{p,q})^{(r)}}$ for any $C\in(\ell^{p,q})^{(r)}$. One has

$$\Vert f_{n}-f_{1}-TZ \Vert _{\mathrm{inf}}= \Biggl\Vert T \Biggl(\sum_{k=n+1}^{\infty}C_{k} \Biggr) \Biggr\Vert _{\mathrm{inf}}\leq \Biggl\Vert \sum _{k=n+1}^{\infty}C_{k} \Biggr\Vert _{(\ell ^{p,q})^{(r)}}\leq\sum_{k=n+1}^{\infty} \Vert C_{k} \Vert _{(\ell^{p,q})^{(r)}}\rightarrow0, $$

when $n\rightarrow\infty$. Therefore, $\operatorname{Ran}(T)$ is closed. Since $V_{p,q}(\Theta)=\operatorname{Ran}(T)$, one sees that $V_{p,q}(\Theta)$ is closed.

$\mbox{(i)}\Rightarrow\mbox{(ii)}$:

Similarly to [1, Proof of $\mbox{(i)}\Rightarrow\mbox{(iii)}$], one can prove $\mbox{(i)}\Rightarrow\mbox{(ii)}$ by using $\mathcal{L}^{\infty,\infty}\subset\mathcal {L}^{\infty}$, and substituting $L^{p,q}$, $\mathcal{L}^{\infty ,\infty}$, Proposition 2.1 and Lemma 2.5 for $L^{p}$, $\mathcal{L}^{\infty}$, Lemma 1 and Lemma 3 in [1], respectively.

$\mbox{(ii)}\Rightarrow\mbox{(iv)}$:

Assume that $h_{\lambda}(\omega)$, $P_{\lambda}(\omega)$ and $\widehat{\Psi}_{1,\lambda}(\omega)$ are as in Proposition 2.2. Define

3.2 $$D_{\lambda }(\omega )=\bar{P_{\lambda }{\left(\omega \right)}^{T}}\left(\begin{array}{cc} {\left(\omega \right)}^{-1} & 0\\ 0 & I \end{array}\right)P_{\lambda }(\omega )H_{\lambda }(\omega ).$$

Here $H_{\lambda}(\omega)$ is a function with period 2*π* which satisfies $\operatorname{supp}H_{\lambda}\subset B(\eta_{\lambda },\delta_{\lambda})+2\pi \mathbb{Z}^{d+1}$ and $H_{\lambda}(\omega)=1$ on $\operatorname{supp}h_{\lambda}$. Thus $D_{\lambda}\in(\mathcal{WC})^{(r\times r)}$. Let $\Psi=(\psi _{1},\psi_{2},\ldots,\psi_{r})^{T}$ be defined by

3.3 $$\begin{aligned} \widehat{\Psi}(\omega)=\sum_{\lambda\in\Lambda}h_{\lambda }( \omega)D_{\lambda}(\xi)\widehat{\Theta}(\omega). \end{aligned}$$

Then, by Lemma 2.4, one has $\Psi\in\mathcal {L}^{\infty,\infty}$. For any $f\in V_{p,q}(\Theta)$, using the definition of $V_{p,q}(\Theta)$, there exists a distribution $A(\omega )\in(\mathcal{WC}^{p,q})^{(r)}$ with period 2*π* which satisfies $\hat{f}(\omega)=A(\omega)^{T}\widehat{\Theta}(\omega)$. Putting

$$g=\sum_{j=1}^{r}\sum _{k\in \mathbb{Z}^{d+1}}\bigl\langle f,\psi_{j}(\cdot -k)\bigr\rangle \theta_{j}(\cdot-k). $$

By the periodicity of $h_{\lambda}(\omega) $ and $D_{\lambda}(\omega )$, (3.2), (3.3) and Proposition 2.2, one has

$$\begin{array}{rcl} \hat{g}(\omega ) & = & A{\left(\omega \right)}^{T}[\hat{\Theta },\hat{\Psi }](\omega )\hat{\Theta }(\omega )\\ & = & \sum_{\lambda \in \Lambda }A{\left(\omega \right)}^{T}P_{\lambda }{\left(\omega \right)}^{-1}\\ & & \times \left(\begin{array}{cc} \left(\omega \right) & 0\\ 0 & 0 \end{array}\right)\left(\begin{array}{cc} {\left(\omega \right)}^{-1} & 0\\ 0 & I \end{array}\right)\left(\begin{array}{c} {\hat{\Psi }}_{1,\lambda }(\omega )\\ 0 \end{array}\right)h_{\lambda }(\omega )\\ & = & \sum_{\lambda \in \Lambda }A{\left(\omega \right)}^{T}P_{\lambda }{\left(\omega \right)}^{-1}\left(\begin{array}{c} {\hat{\Psi }}_{1,\lambda }(\omega )\\ 0 \end{array}\right)h_{\lambda }(\omega )\\ & = & \sum_{\lambda \in \Lambda }A{\left(\omega \right)}^{T}P_{\lambda }{\left(\omega \right)}^{-1}P_{\lambda }(\omega )\hat{\Theta }(\omega )h_{\lambda }(\omega )\\ & = & \sum_{\lambda \in \Lambda }A{\left(\omega \right)}^{T}\hat{\Theta }(\omega )h_{\lambda }(\omega )\\ & = & A{\left(\omega \right)}^{T}\hat{\Theta }(\omega )\\ & = & \hat{f}(\omega ). \end{array}$$

Thus $\hat{f}(\omega)=\hat{g}(\omega)$. Therefore $f=g$, namely

$$f=\sum_{j=1}^{r}\sum _{k\in \mathbb{Z}^{d}}\bigl\langle f,\psi_{j}(\cdot -k)\bigr\rangle \theta_{j}(\cdot-k). $$

Similar arguments show that

$$f=\sum_{j=1}^{r}\sum _{k\in \mathbb{Z}^{d}}\bigl\langle f,\theta_{j}(\cdot -k)\bigr\rangle \psi_{j}(\cdot-k). $$

## Concluding remarks

In this paper, we study the closedness of shift invariant subspaces in $L^{p,q} (\mathbb{R}^{d+1} )$. We first define the shift invariant subspaces generated by the shifts of finite functions in $L^{p,q} (\mathbb{R}^{d+1} )$. Then we give some necessary and sufficient conditions for the shift invariant subspaces in $L^{p,q} (\mathbb{R}^{d+1} )$ to be closed.

However, in this paper, we only consider the closedness of shift invariant subspace of $L^{p,q} (\mathbb{R}^{d+1} )$. Studying the $L^{p,q}$-frames in a shift invariant subspace of mixed Lebesgue $L^{p,q}(\mathbb{R}^{d})$ is the goal of future work.
